# The Use of Polliniferous Resources by *Melipona capixaba*, an Endangered Stingless Bee Species

**DOI:** 10.1673/031.012.14801

**Published:** 2012-12-20

**Authors:** Bruna Danielle Vieira Serra, Cynthia Fernandes Pinto da Luz, Lucio Antonio de Oliveira Campos

**Affiliations:** ^1^ Departamento de Biologia Animal, Programa de Pós-graduação em Entomologia, Universidade Federal de Viçosa, Av. P.H. Rolfs, s/n, Viçosa, Minas Gerais, 36570-000, Brazil; ^2^ Instituto de Botânica, Núcleo de Pesquisa em Palinologia, Caixa Postal 3005, São Paulo, São Paulo, 01061-970, Brazil; ^3^ Departamento de Biologia Geral, Universidade Federal de Viçosa, Av. P.H. Rolfs, s/n, Viçosa, Minas Gerais, 36570000, Brazil

**Keywords:** bee-plant relationship, Eucalyptus, foraging, pollen analysis, pollen sources

## Abstract

Pollen types present in samples from corbiculae of *Melipona capixaba* (Moure and Camargo) (Hymenoptera: Apidae: Meliponina) worker bees were analyzed, as well as pollen samples from food pots inside the hives in three sites located at the bees’ original habitat. The aim was to find out the sources used as a trophic resource by this species. The dominant pollen grains in the spectrum of the samples belonged to the families Myrtaceae and Melastomataceae. *Eucalyptus* was the most frequent pollen type in the corbiculae in Conceição do Castelo municipality; *Eucalyptus*, *Myrcia*, and Melastomatacea/Combretaceae in the Fazenda do Estado district; and *Eucalyptus* and *Myrcia* in the São Paulo de Aracê district, both in the Domingos Martins municipality. *Eucalyptus* and Melastomataceae/Combretaceae were the predominant pollen types in the food pots. *Eucalyptus* was the most prevalent type all year round or most of the year. The most common pollen types in the months that *Eucalyptus* was not present or dominant in the samples were of remaining native forest species, “ruderal” (field) plants, fruit-bearing plants, and introduced ornamental plants.

## Introduction

*Melipona capixaba* (Moure and Camargo) (Hymenoptera: Apidae: Meliponina), locally known as uruçu-preta, is an endemic bee to mountainous areas (between 900 and 1000 m.a.s.l.) of the Atlantic Rainforest in southwest Espírito Santo, Brazil (Moure and Camargo 1994; Melo 1996). The biome where this bee occurs is considered one of the twenty five hotspots existing in the world, being the fourth one in endemism rate and among the eight with the highest biological diversity (Myers et al. 2000). The decrease in areas of original habitat and changes in its quality, associated with limited and fragmented distribution of *M. capixaba*, led to its inclusion as vulnerable in the list of Brazilian Endangered Species in 2003 (IPEMA 2007), being the only Brazilian stingless bee with this status. Its reduced occurrence may be evolutionarily linked to local biological characteristics, such as the native flora of the region. The scarcity of data about its biology, ecology, and ethology hinders its conservation. In order to mitigate the risk and vulnerability of the species, it is fundamental to know the botanic taxa important as a trophic resource to the colonies of *M. capixaba*.

Pollen is an energy source for bees and supplies vitamins, minerals, lipids, sugars, and starch and nitrogen compounds, being essential for the growth of the tissues of young individuals (Goodman 2003). Its harvest and storage must be constant because eusocial bee colonies are perennial, populous, and characterized by continuous offspring production. Thus, the maintenance of the hives should be assured in periods of low food availability in the field (Michener 2007). The identification of plant species used as food by bees is made through melissopalinology, which allows the identification of pollen grains based on their morphology. The quantitative and qualitative result constitutes the pollen spectrum, which corresponds to the plants visited by the bees in a certain region (Barth 1989). Pollen analyses are useful for indirect determination of the trophic resources used by bees, helping in the elucidation of their roles as pollinators. The wide spectrum presented by eusocial bees allows their classification as polythetic, because they harvest pollen from several plant species (Ramalho et al. 1989, 1990; Ramalho 2004; Luz et al. 2007, 2010).

The only existing papers in literature about visitation of plant species by *M. capixaba* are assigned to Resende et al. (2008) and Luz et al. (2011). Resende et al. (2008) reported the presence of pollinarium of orchids from tribe Cymbidiae, subtribe Maxillariinae, in the scutellum of this bee. Luz et al. (2011) cited the sources used by *M. capixaba* through the palynological analysis of pollen stored in food pots of eleven colonies in meliponaries from the municipalities of Alfredo Chaves, Domingos Martins, and Venda Nova do Imigrante, indicating *Eucalyptus* and *Tibouchina* as the main pollen sources. Because Luz et al. 2011 sampled only three non-consecutive months in three different years, their results do not provide a detailed picture of the seasonal pattern of pollen collection by *M. capixaba* throughout the year.

The present study addresses this shortcoming. Pollen samples were taken monthly over a full year, both from the corbiculae and from food pots, from different sampling locations, in an effort to more broadly understand the trophic resources utilized by *M. capixaba*. The results will facilitate efforts to preserve the species.

## Materials and Methods

### Habitat

The vegetation in the region includes many endemic species and is part of the Atlantic Rainforest domains (Rizzini 1992). It was classified by Veloso et al. (1991) as a dense, ombrophilous forest. The three sites sampled had remaining native vegetation nearby, were surrounded by *Eucalyptus* plantations, varied agricultural crops (coffee, garlic, carrot, maize, sweet pepper, tomato, etc.), pastures and domestic yards with fruit-bearing plants (avocado, guava, jabuticaba, orange, lemon, etc.), and ornamental plants. A listing of the native flora preserved in the State Park of Pedra Azul, in Domingos Martins, is available at CEPEMAR (2004). The municipalities are included in Serra Capixaba relief unit, which contains huge massifs with elevations exceeding 1200 m.a.s.l. This unit is inserted in the natural zone of cold, rough, and rainy lands, with a minimum average temperature of the coldest month ranging from 7.3 to 9.4° C and a maximum average temperature of the hottest month ranging from 25.3 to 27.8° C (INCAPER 1999). The rainy period occurs between October and April, and the annual average rainfall ranges from 943 to 1906 mm. The average relative humidity is 86.3% (CEPEMAR 2004).

### Study sites

**Figure 1.**
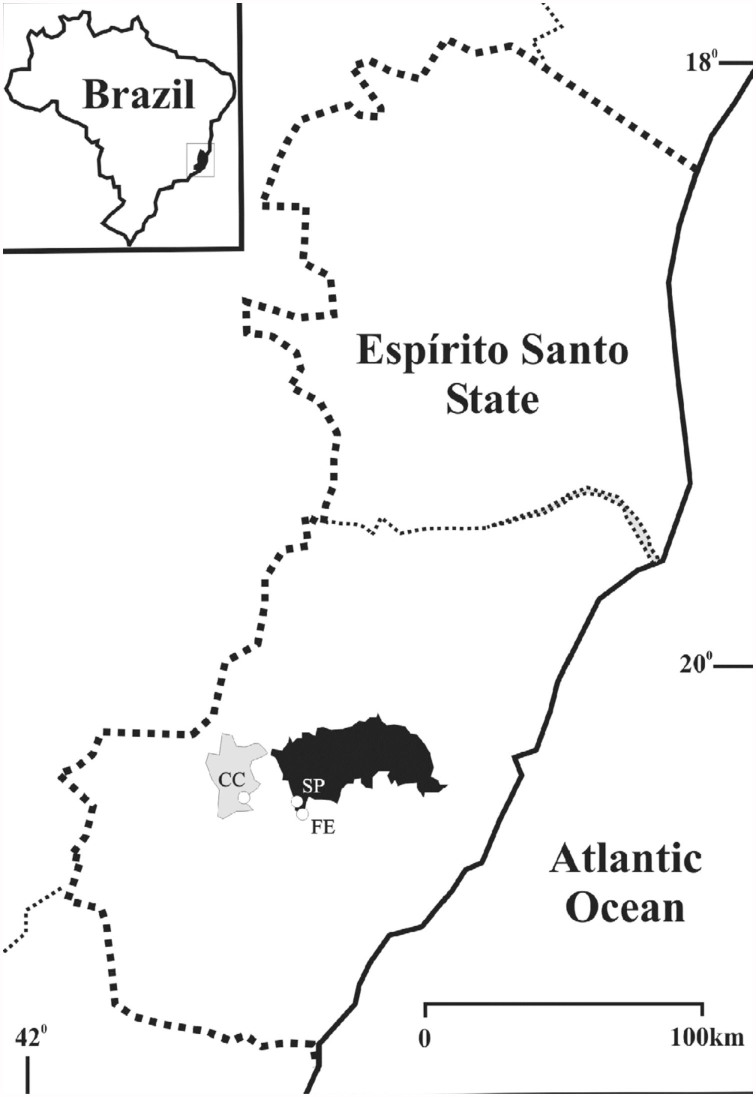
Municipalities where the pollen samples from corbiculae and food pots of hives of *Melipona capixaba* were collected, Espírito Santo, Brazil. (CC: Conceição do Castelo municipality, FE: State Treasury district in Domingos Martins municipality, SP: Aracê district of São Paulo in Domingos Martins municipality). High quality figures are available online.

Monthly collections were made for one year in meliponaries in three sites in the state of Espírito Santo: Conceição do Castelo municipality (CC) (June to September 2009) (S 20° 21′ 48.4″; W 41° 14′ 44.4″; 627 m.a.s.l.), Fazenda do Estado (FE) (October 2009 to May 2010) (S 20° 22′ 12.4″; W 41° 03′ 50.3″; 900 m.a.s.l.), and São Paulo de Aracê (SP) (June 2009 to May 2010) (S 20° 25′ 58.8″; W 41° 02′ 05.3″; 1021 m.a.s.l.), the last two sites being districts of Domingos Martins municipality (Figure 1). A meliponary in CC was replaced by one in FE in October 2009 due to the location of its colonies in an altitude lower than the one where wild hives were found. The difficulty in finding beekeepers that allowed the use of their colonies in research and the rarity of the species in nature resulted in the use of colonies in CC at the beginning.

### Pollen sampling from corbiculae

Three colonies were sampled at each site except SP, at which only two colonies were sampled because one died in September 2009. The reduced number of sampled colonies was due to the rarity of the species and the restriction on the use of hives for data collection by beekeepers. The collections were made on three consecutive days every month, and samples were taken every two hours, from 06:00 to 16:00. To this purpose, the entrance of the hives were closed for ten minutes and pollen loads were removed from the corbiculae of bees that were returning from the field to the hive. 5321 pollen samples from corbiculae of *M. capixaba* were collected. 749 samples were collected in CC, 2213 in FE, and 2359 in SP. Each collected sample corresponded to the pollen load of a single bee. Microscope slides were prepared with content of each sample according to Maurizio and Louveaux (1965). Each pollen sample was dissolved in 20 mL of distilled water. The sediment was re-suspended in a 1:1 glycerol: H_2_O mixture and subsequently mounted with glycerine-jelly on microscope slides sealed with paraffin. For each meliponary, corbiculae samples obtained each month were pooled and used to calculate the mean percentage of each pollen type collected during that month.

### Pollen sampling stored in food pots

The collection of pollen stored in food pots was done to sample the pollen types used by *M. capixaba* during periods between the sampling from corbiculae. This sampling was only taken from the three colonies in FE (from July 2009 and June 2010) because they were the only ones authorized by the beekeepers to be handled. In this procedure, the hives were open once each month, in the same days of pollen collection from corbiculae. The food pots were sampled through the insertion of a plastic straw, which collected pollen accumulated throughout the vertical extension. A sample was taken from each food pot of the three hives. 270 pollen samples were collected from food pots inside the hives in FE. Microscope slides were prepared with the content of each sampled food pot according to Maurizio and Louveaux (1965) without the use of acetolysis. For the analyses of pollen types found in food pots, the samples were grouped per month.

### Melissopalynological analysis

The qualitative analysis took into consideration the pollen types identified because it is often not possible to specify genus or species from the pollen morphology (Barth 1989). The samples were analyzed quantitatively by counting 500 pollen grains per sample from all microscopical field, which were randomized in order to calculate the relative frequencies of dominant pollen type (PD: > 45% of the total pollen grains), accessory pollen (PA: 15–45%), and isolated pollen (PIi: 3–15% and PIo: < 3%) (Barth 1989). The identification of pollen types was based on comparisons with reference pollen types from the Botanic Institute of São Paulo and palynological catalogues (Barth 1970a, 1970b, 1970c, 1970d; Melhem et al. 1984; Barth 1989; Roubik and Moreno 1991). Comparisons with floristic inventories of Espírito Santo were made for determining the plant species visited by the bees (CEPEMAR 2004; Jesus and Rolim 2005; Rolim et al. 2006; SEMA 2008). Pollen grains were photographed using a Zeiss Primo Star photomicroscope, which was linked to a video camera and microcomputer with the software Axiovision.

## Results

### Pollen from corbiculae

From the total, 5249 (98.65%) were loads of monofloral pollen (pollen type count larger than 90%) and 72 (1.35%) were loads of heterofloral pollen (with several pollen types). A total of 56 pollen types were observed in the samples, of which 52 genera and 25 families were recognized, as well as a type identified as a Monocotyledon. 26 pollen types were observed in the samples of CC, 38 in the samples of FE, and 42 in the samples of SP. Some pollen types only occurred in CC (*Calliandra*, *Eugenia*, *Eupatorium*, *Persea*, and *Typha*), others only in FE (*Acnistus*, *Anadenanthera*, *Cordia*, *Faramea*, *Mimosa caesalpiniaefolia*, *Myrsine*, *Phthirusa*, and *Trema micrantha*), and others only in SP (*Aparisthmium*, *Commelina virginica*, *Coussarea*, *Elephantopus*, *Hyptis*, Monocotyledon, *Schizolobium parahybum*, *Sida*, *Stylosanthes*, *Thunbergia*, and *Zanthoxylum*) (Tables 1 and 2). The families that presented the highest number of pollen types were Fabaceae (13), Asteraceae (5), Euphorbiaceae (4), Rubiaceae (4), Myrtaceae (3), Sapindaceae (3), and Solanaceae (3).

The dominant pollen type in CC was *Eucalyptus*. The types *Myrcia*, *Cupania*, and *Baccharis* had high percentages. Other pollen types with low percentages characterized the native vegetation (*Alchornea*, *Eugenia*, *Euterpe/Syagrus*, *Guapira*, *Machaerium*, Melastomataceae/Combretaceae, *Mimosa verrucosa*, *Paullinia*, *Senna*, *Serjania*, and *Tapirira*), ruderal plants (*Crotalaria*, *Eupatorium*, *Typha*, and *Vernonia*), cultivated fruit bearing plants (*Citrus*, *Coffea*, and *Persea*), cultivated ornamental plants (*Calliandra* and *Montanoa*), and plants that can present different habitats *(Solanum*) (Tables 1 and 3).

The dominant pollen types in FE were *Eucalyptus*, Melastomataceae/Combretaceae, and *Myrcia*. The types *Alchornea*, *Coffea*, *Heisteria*, *Mimosa caesalpiniaefolia*, and *Solanum* were classified as high percentage. Low percentages were observed for pollen types of native vegetation (*Acacia*, *Anadenanthera*, *Bauhinia forficata*, *Cupania*, *Euterpe/Syagrus*, Faramea, Inga, *Parabignonia*, *Machaerium*, *Mimosa verrucosa*, *Myrsine*, *Paullinia*, *Phthirusa*, *Piptadenia communis*, *Psychotria*, *Senna*, *Serjania*, *Tapirira*, and *Trema micrantha*), ruderal plants (*Crotalaria*, *Croton*, and *Vernonia*), cultivated fruit-bearing plants (*Citrus*), cultivated ornamental plants (*Acalypha*, *Acnistus*, *Cordia*, and *Lantana*) and plants of varied habitats (Solanaceae) (Tables 1–3).

The dominant pollen types in SP were *Eucalyptus* and *Myrcia*. The types *Crotalaria*, Melastomataceae/Combretaceae, *Senna*, and *Solanum* were classified as high percentage. Other pollen types with lower percentages characterized the native vegetation (*Acacia*, *Alchornea*, *Aparisthmium*, *Bauhinia forficata*, *Coussarea*, *Cupania*, *Euterpe/Syagrus*, *Guapira*, *Heisteria*, *Inga*, *Parabignonia*, *Machaerium*, *Paullinia*, *Piptadenia communis*, *Psychotria*, *Schizolobium parahybum*, *Serjania*, *Struthanthus*, *Tapirira*, and *Zanthoxylum*), ruderal plants (*Baccharis*, *Croton*, *Elephantopus*, *Hyptis*, *Sida*, and *Vernonia*), cultivated fruit bearing plants (*Citrus* and *Coffea*), cultivated ornamental plants (*Acalypha*, *Commelina virginica*, *Lantana*, *Montanoa*, and *Thunbergia*), cultivated plants in pastures (*Stylosanthes*), and plants that can occur in several habitats (Monocotyledon and Solanaceae) (Tables 1–3).

### Pollen stored in food pots

A total of 42 pollen types were observed in the samples, of which 39 genera and 21 families were recognized, as well as a type identified as a Monocoliledon. The pollen types identified in the samples from food pots that did not occur in the samples from corbiculae were *Caesalpinia* (Fabaceae) and *Piper* (Piperaceae). Monofloral samples were not identified. The dominant pollen types were *Eucalyptus* and Melastomataceae/Combretaceae. The types *Euterpe/Syagrus*, *Heisteria*, *Myrcia*, *Senna*, and *Solanum* were classified as high percentage. Low percentages were observed for pollen types of native vegetation (*Acacia*, *Alchornea*, *Aparisthmium*, *Bauhinia forficata*, *Caesalpinia*, *Cupania*, *Faramea*, *Guapira*, *Heisteria*, *Inga*, *Parabignonia*, *Machaerium*, *Mimosa caesalpiniaefolia*, *Mimosa verrucosa*, *Paullinia*, *Phthirusa*, *Piper*, *Piptadenia communis*, *Schizolobium parahybum*, *Serjania*, *Tapirira*, and *Zanthoxylum*), ruderal plants (*Baccharis*, *Crotalaria*, *Eupatorium*, *Hyptis*, *Sida*, and *Vernonia*), cultivated fruit bearing plants (*Coffea* and *Persea*), cultivated ornamental plants (*Acalypha*, *Montanoa* and *Thunbergia*), and plants that may occur in several habitats (Monocotyledon and Solanaceae) (Tables 1–3).

## Discussion

The dominant pollen types in the spectrum of the samples of *M. capixaba* belonged to the families Myrtaceae and Melastomataceae. The same result was found by Luz et al. (2011) in the analysis of pollen stored in hives of *M. capixaba* in four different meliponaries in the region. These families are among the most important pollen sources for the genus *Melipona* (Ramalho et al. 1989, 1990; Carvalho et al. 2001; Alves et al. 2006; Costa and Martins 2006; Oliveira et al. 2009). Myrtaceae and Melastomataceae have their center of dispersion coincident with the distribution of this bee genus, exclusively neotropical (Silveira et al. 2002), which may be related to the intensive use of these families by *Melipona* spp. (Ramalho et al. 1989, 1990). Additionally, flowers of several species of Myrtaceae and Melastomataceae present poricidal anthers, which require specialized behavior of their visitors to release pollen grains (Ramalho et al. 1989; Proença and Gibbs 1994). The pollen of these anthers is extracted only by vibration of flight muscles (“buzz pollination”), a behavioral specialization present in *Melipona* spp. and absent in several other bee species (Michener 2007).

The predominance of these families is also related to their mass blooming with a duration of several days (Proença and Gibbs 1994; Meyer 1998; Danner et al. 2010), favoring the visitation of *Melipona* spp., which forage primarily in dense flower resources rich in pollen and/or nectar in order to maintain their populous colonies (Roubik 1993; Ramalho 2004). Mass blooming stimulates fidelity and floral constancy, favoring cross-pollination, since bees tend to forage a specific flower type instead of several types. Foraging a single plant species saves energy, since it will not be necessary to use different harvest and flower handling mechanisms in successive trips (Ramalho et al. 1994; Michener 2007).

*Eucalyptus*, the dominant pollen type in the samples of *M. capixaba* from the three sites sampled all year round or for most of the year, owes its prevalence to the presence of reforesting areas of *Eucalyptus* spp. next to the sites where the colonies were located. These plantations made by rural producers in small properties in the region are destined to be utilized for their wood, especially in pallets production (wooden bases used to move loads), house construction, and charcoal production.

The use of *Eucalyptus* floral resources has already been observed in several species of *Melipona*. The presence of *Eucalyptus* nectar and pollen was reported as dominant or important in the pollen spectrum of honeys and pollen loads, being considered a good option for bee pasture (Kleinert-Giovannini and Imperatriz-Fonseca 1987; Ramalho et al. 1990, 1994; Carvalho et al. 2001; Costa and Martins 2006; Oliveira et al. 2009). However, the nutritional quality of their grains should be evaluated since some species of *Eucalyptus* can present pollen with low concentrations of lipids and essential amino acids such as isoleucine and tryptophan, negatively affecting the longevity of bees and leading to a reduction of their populations (Bell et al. 1983; Somerville 2001; Manning et al. 2007). As *M. capixaba* is a vulnerable species in the list of Brazilian Endangered Fauna, the heavy dependence on *Eucalyptus* should be treated carefully.

Although dominant in the samples as a whole, *Eucalyptus* was not collected in some months, or was collected but not dominant. When *Eucalyptus* was not collected or was not dominant, pollen types of wild plant species were preponderant, assuring the visitation of *M. capixaba* to several plants of the native forest, as well as to ruderal, fruit-bearing, and ornamental plants. Luz et al. (2011) confirmed *M. capixaba* preferred pollen of *Tibouchina*, a native tree in this forest, when distant from the areas of *Eucalyptus* plantations. Analyzing the three sites, it can be concluded that *Eucalyptus* presented flowering all year round. Thus, the dominance of pollen types of wild, ruderal, fruit-bearing, and ornamental plants in some months seems to be due to the higher attraction that these species exerted on *M. capixaba* in certain periods of the year. The use of wild plants by the bees factors into the importance of preserving Atlantic Rainforest native flora components for the survival of colonies in certain periods of the year.

Among pollen samples from food pots, only *Caesalpinia* and *Piper* were not found in the samples from corbiculae. The presence of *Piper* was observed at a low percentage in pollen samples collected from hives of *M. capixaba* in Venda Nova do Imigrante and Domingos Martins municipalities (Luz et al. 2011). The samples from the corbiculae presented a higher number of pollen types used by *M. capixaba* than the samples from the food pots. This was possibly caused by the smaller number of analyzed pollen samples in the food pots, as the number of observed floral interactions decreased just by retrieving of pollen of corbiculae from some bees returning from the field. As the pollen from the food pots is analyzed, it is observed that pollen grains from new plant species are identified as the storage period is much longer compared to sporadic collection of bees (during three consecutive days, e.g.). However, the integration of methods enlarged the richness of the spectrum correspondent to the sources used by *M. capixaba*.

The main trophic resources used by *M. capixaba* belong to the families Myrtaceae and Melastomataceae, and *Eucalyptus* features as an important plant for this bee's diet throughout the year. The abundance of *Eucalyptus* bee pasture should be considered carefully because it is not known what effects the components of its pollen may have on the development of *M. capixaba* individuals and thus on the maintenance of their populations in natural environments. Pollen analysis showed the primary autochthonous plants in which *M. capixaba* searched for its pollen resources, indicating that in the presence of native floral resources this bee visits and collects efficiently these original pollen sources. The results of this work could provide important information for future research on pollination ecology and biology of these plants, with the intention to preserve *M. capixaba*.

**Table 1.**
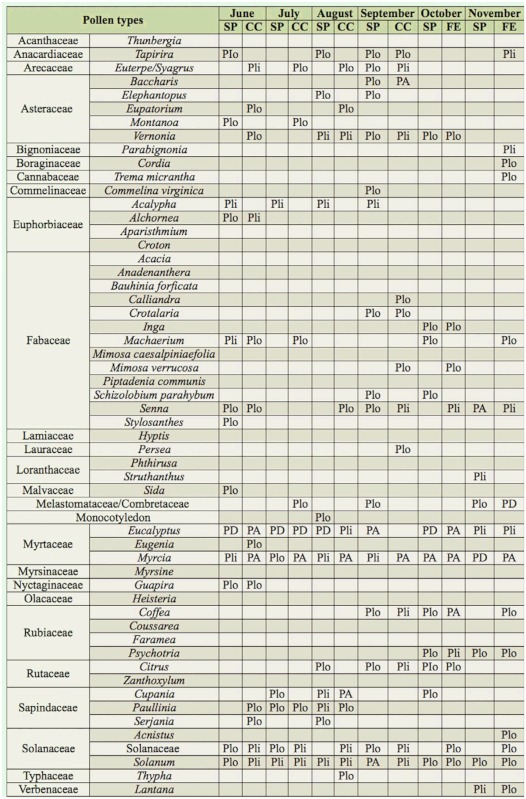
Pollen types observed in pollen loads from corbiculae of *Melipona capixaba* in Conceição do Castelo municipality (CC) and in the districts of Fazenda do Estado (FE) and São Paulo de Aracê (SP), in Domingos Martins municipality from June (Jun) to November (Nov) 2009 in Espírito Santo, Brazil. PD = dominant pollen (> 45%); PA = accessory pollen (15–45%); Pli = important isolated pollen (3–14%); Plo = occasional isolated pollen (< 3%).

**Table 2.**
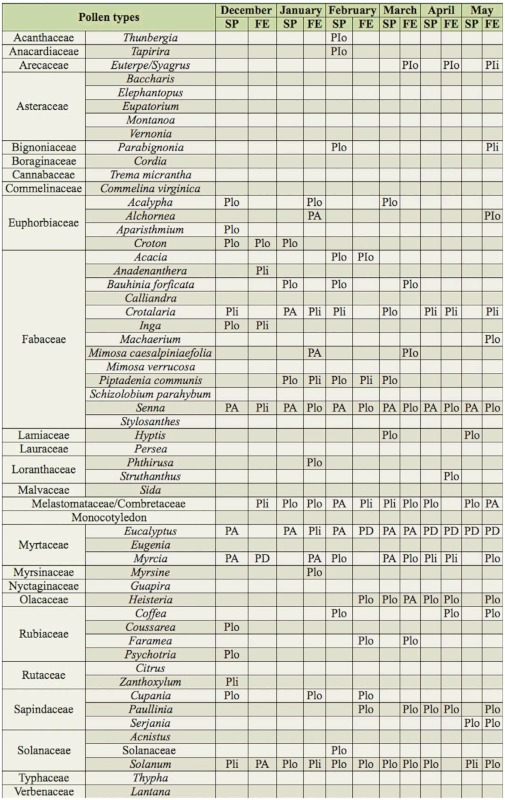
Pollen types observed in pollen loads from corbiculae of *Melipona capixaba* in the districts of Fazenda do Estado (FE) and São Paulo de Aracê (SP), in Domingos Martins municipality, Espírito Santo, Brazil, from December (Dec) 2009 to May (May) 2010. PD = dominant pollen (> 45%); PA = accessory pollen (15–45%); Pli = important isolated pollen (3–14%); Plo = occasional isolated pollen (< 3%).

**Table 3.**
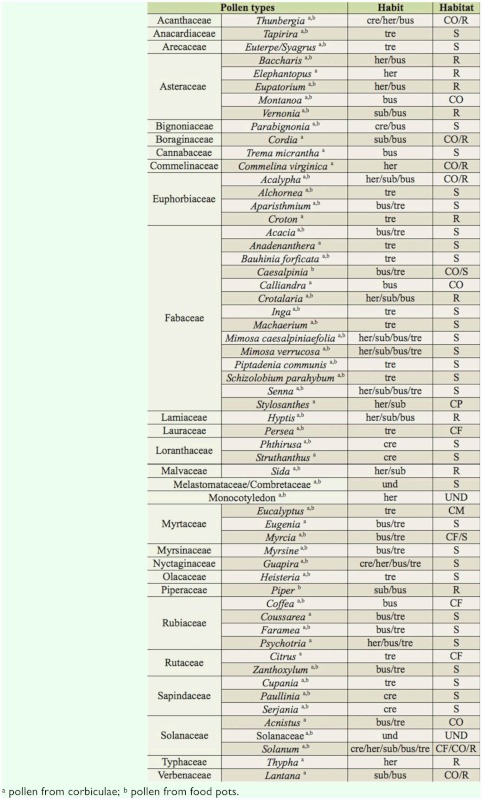
Grand compilation of pollen types identified in pollen loads from corbiculae and food pots, with data on habit and habitat. Data collected from hives of *Melipona capixaba* in Conceição do Castelo municipality and in the districts of Fazenda do Estado and São Paulo de Aracê, in Domingos Martins municipality, Espírito Santo, Brazil. (Habit: *Cre* = creeper; *Her* = herbaceous; *Sub* = subshrub; *Bus* = bush; *Tre* = tree, *Und* = undetermined. Habitat: *CF* = cultivated fruit-bearing; *CW* = cultivated woody; *CO* = cultivated ornamental; *CP* = cultivated in pastures; *R* = ruderal; *W* = wild; *UND* = undetermined).

## Abbreviations

CC,: Conceição do Castelo municipality;
FE,: Fazenda do Estado;
SP,: São Paulo de Aracê
